# Role of Proteases in Extra-Oral Digestion of a Predatory Bug, *Andrallus spinidens*

**DOI:** 10.1673/031.012.5101

**Published:** 2012-04-11

**Authors:** Arash Zibaee, Hassan Hoda, Fazeli-Dinan Mahmoud

**Affiliations:** ^1^ Department of Plant Protection, College of Agriculture, University of Guilan-Rasht, 41635-1314, Iran; ^2^ Biological Control Department, National Institite of Plant Protection, Amol, Iran; ^3^ Department of Plant Protection, College of Agriculture and Natural Resources, University of Tehran, Karaj 31584, Iran

**Keywords:** biochemical characterization, indigenous inhibitors, proteolytic activity, synthetic inhibitors

## Abstract

Roles of salivary proteases in the extra-oral digestion of the predatory bug, *Andrallus spinidens* Fabricius (Hemiptera: Pentatomidae) were studied by using 2% azocasein as a general substrate and specific protease substrates, as well as synthetic and endogenous inhibitors. It was found that salivary glands of *A. spinidens* have two anterior, two lateral, and two posterior lobes. Azocasein was used to measure the activity of general proteases in the salivary glands using different buffer solutions. The enzyme had the highest activity at pH 8. General protease activity was highest at 40 °C and was stable for 6–16 hours. The use of specific substrates showed that trypsin-like, chymotrypsin-like, aminopeptidase, and carboxypeptidase are the active proteases present in salivary glands, by the maximum activity of trypsin-like protease in addition to their optimal pH between 8–9. Ca^2+^ and Mg^2+^ increased proteolytic activity about 216%, while other ions decreased it. Specific inhibitors including SBTI, PMSF, TLCK, and TPCK significantly decreased enzyme activity, as well as the specific inhibitors of methalloproteases including phenanthroline, EGTA, and TTHA. Extracted endogenous trypsin inhibitors extracted from potential prey, *Chilo suppressalis, Naranga aenescens, Pieris brassicae, Hyphantria cunea*, and *Ephestia kuhniella*, had different effects on trypsin-like protease activity of *A. spinidens* salivary glands. With the exception of *C. suppressalis*, the endogenous inhibitors significantly decreased enzyme activity in *A. spinidens*.

## Introduction

The majority of terrestrial predaceous arthropods use extra-oral digestion as a tool of utilizing relatively large preys. These predators obtain prey extraction and nutrient concentration by refluxing or nonrefluxing application during injection of hydrolytic enzymes (Cohen 1995). The advantages of this process are considered ecologically as an abbreviation of handling time and an increase in the nutrient density of consumed food, allowing small predators to consume relatively large prey. The basis of extra-oral digestion is a highly coordinated combination of biochemical, morphological, and behavioral adaptations that vary with different taxa (Cohen 1995).

The predatory bug, *Andrallus spinidens* Fabricius (Hemiptera: Pentatomidae) is a potential biological agent of caterpillars that has been widely distributed around the world (Thomas 1994). It has been reported as a potential predator of rice pests in India, Malaysia, and Iran (Nageswara Rao 1965, Manley 1982, Mohaghegh and Najafi 2003). Both nymphs and adults fed on several caterpillars such as *Chilo suppressalis, Naranga aenescens*, and *Helicoverpa armigera* in the rice fields of northern Iran (Mohaghegh and Najafi 2003). Najafi-Navaee et al. (1998) reported that *A. spinidens* is a specific caterpillar feeder in the rice fields of northern Iran that has five generations per year and plays a critical role in regulation of rice pest populations.

Proteases are one the most important digestive enzymes that have crucial roles in converting proteins to oligo- and di-peptides. These enzymes are classified based on amino acids in their active site and the site of activity on protein molecules (Terra and Ferriera 2005). Proteinases (Endopeptidases) are responsible for initial digestion of proteins by breaking internal bonds. Because of variance in peptide chains, different proteinases are necessary to break these bonds. These proteinases have been classified to three main subclasses according to their active site, namely serine, cysteine, and aspartic proteinases (Terra and Ferriera 2005). In each of these subclasses, there are several proteinases differing in substrate specificities. The oligopeptides resulting from proteinase action are attacked from the N-terminal end by aminopeptidases and from the C-terminal end by carboxypeptidases; both enzymes liberate one amino acid residue at each catalytic step (Terra and Ferriera 2005).

To our knowledge, such proteolytic diversity has not been identified in *A. spinidens*; however, results on *Podisus maculiventris*, and some other predaceous bugs, demonstrated that protease activity in *P. maculiventris* salivary secretions was mainly based on serine proteases, whereas cysteine proteases and exopeptidases are predominant in the gut (Stamopoulos et al. 1993; Bell et al. 2005, Alvarez-Alfageme et al. 2007, Pascual-Ruiz et al. 2009). Edwards (1961) characterized an alkaline endopeptidase in the saliva of *Platymeris rhadamanthus* using azocasein as substrate. Cohen (1989) studied the salivary protease efficiency of *Geocoris punctipes*. The amino acid profiles of intact aphids compared with others differed somewhat, indicating that some proteins were inaccessible or indigestible to the predators. Ingestion efficiency averaged 64.6%, and the approximate digestibility of ingested material was > 90%. Proteases have a crucial role in extra-oral digestion of predaceous bugs showing their possible role in prey preference.

Also, extra-oral digestion involves an integration of biological, biochemical, physiological, morphological, and behavioral adaption (Cohen 1998). Hence, the aims of this study were: (i) determination of general and specific proteolytic activity; (ii) effects of pH and temperature; (ii) effects of various ions; (iii) effects of general and specific inhibitors; and (iv) effects of endogenous trypsin inhibitors from *Chilo suppressalis* Walker (Lepidoptera: Crambidae), *Naranga aenescens* Moore (Lepidoptera: Noctuidae), *Pieris brassicae* L. (Lepidoptera: Pieridae), *Hyphantria cunea* Drury (Lepidoptera: Noctuidae), and *Ephestia kuhniella* Zeller (Lepidoptera: Pyralidae) on the proteolytic activity in the salivary glands of *A. spinidens*.

## Materials and Methods

### *A. spinidens* rearing

A colony of *A. spinidens* was established by adults collected from harvested rice fields in Amol, Mazandaran, northern Iran, in late September 2010. Insects were reared on late instars of *Galleria mellonella* L. (Lepidoptera: Pyralidae) as prey and provided with wet cotton plugs fitted into small plastic dishes (2.5 cm diameter) as moisture sources.

### *A. spinidens* dissection and sample preparation

The method described by Cohen (1993) was used to dissect the adults of *A. spinidens*. Adults were randomly selected and their salivary glands were removed by dissection using a stereomicroscope in ice-cold distilled water. Pronotom was gently separated and other tissues such as fat bodies and muscles were carefully removed from the surface of salivary glands. The salivary glands were then cut from the body, rinsed in 1 mL of ice-cold distilled water. To obtain appropriate samples, five salivary glands were placed in one Eppendorff tubes (www.eppendorf.com) containing 1 mL of distilled water. Tissues were ground in a homogenizer and then centrifuged at 12,000 rpm for 15 min at 4 °C. Supernatant was carefully removed and transferred to new tubes and stored at -20 °C for no more than one week until the onset of the experiments.

### General proteolytic activity

Four 2% azocaseinin buffer solutions including universal buffer (2mM, containing succinate, glycine, and 2- morpholinoethanesulfonic acid; pH range 3–14; Frugoni 1957), Tris-base (2 mM, pH range 3–10), Citrate-phosphate (2 mM, pH range 3– 8), and phosphate buffer (2 mM, pH range 6.5–8.5) were used to determine the general proteolytic activity in salivary glands of adult *A. spinidens*. General proteolytic activity was measured using 2% azocasein based on a method described by Elpidina et al. (2001). The reaction mixture consisted 100 µL of appropriate buffer solutions, 50 µL 2% azocasein, and 20 µL enzyme solution. After incubation at 37 °C for 60 min, proteolysis was stopped by addition 150 µL of 10% trichloroacetic acid (TCA). Precipitation was achieved by cooling at 4 °C for 120 min, and centrifugation at 10000 × g for 10 min. An equal volume of 2 M NaOH was added to the supernatant, and absorbance was recorded at 450 nm. Blank solution consisted all mentioned portions except for enzyme solution.

### Determination of optimal temperature (°C) on general proteolytic activity and stability

Temperatures from 15–80 °C were used to find optimal temperature for general proteolytic activity in salivary glands when 2% azocasein was used as the substrate. The reaction mixtures were similar to those described earlier, but the buffer solution used was the universal buffer at pH = 8. The incubation temperature ranged from 15–80 °C. To determine stability of the enzyme at optimal temperature, incubation times ranged from 1 to 120 hours in the temperature that enzyme showed the highest activity.

### Specific proteolytic activity *Serine proteolytic activity*

Trypsin-, chymotrypsin-, and elastase-like activities (as three subclasses of serine proteases) were assayed using a concentration 1mM of BApNA (Nabenzoyl- L-arginine-p- nitroanilide), 1 mM SAAPPpNA (N-succinylalanine-alanine-proline-phenylalanine-pnitroanilide), and 1 mM SAAApNA (N-succinyl-alanine-alanine- alanine-p-nitroanilide) as substrates, respectively. The reaction mixture consisted 50 µL of universal buffer (pH = 8), 10 µL of each substrate, and 5 µL of enzyme solution. This pH is considered optimal for these substrates. The reaction mixture was incubated at 37 °C for a period ranging from 5–60 min before adding 10% TCA to terminate the reaction. The absorbance of the resulting mixture was then measured spectrophotometrically at 405 nm by p-nitroaniline release. To prove the specific proteolytic activity, a negative control was provided for each substrate separately containing all mentioned components except for enzyme pre-boiled at 100 °C for 30 min (Oppert et al. 1997).

***Cystein protease***. For cysteine protease assay, benzyloxycarbonyl-Arg-ArgpNA was used as the substrate. Hydrolysis of the 1 mM final concentration of the substrates was determined by measuring the absorbance by p-nitroaniline after 30 min of incubation. The absorbance of different concentrations of pnitroaniline was read at 405 nm to find the extinction coefficient for specific activity calculation. To show the specific proteolytic activity, a negative control was provided for each substrate separately containing all mentioned components except for enzyme pre-boiled at 100 °C for 30 min (Opert et al. 1997).

***Exopetidase activity***. Leucine p-nitroanilide (LpNA) (1 mM) was used to find aminopeptidase activity in the salivary glands. The reaction mixture consisted 80 µL of universal buffer (pH 8), 10 µL of substrate and 5 µL of enzyme solution. The reaction mixture was incubated at 37 °C for a period from 5–60 min before adding 10% TCA to terminate the reaction. The absorbance of the resulting mixture was then measured spectrophotometrically at 340 nm by pnitroaniline release (Opert et al. 1997).

N-(3-(2-furyl) acryloyl)-L-phenylalanyl-Lphenylalanine was used to measure carboxypeptidase activity in the salivary glands. The reaction mixture consisted 80 µL of universal buffer (pH 8), 10 µL of substrate, and 5 µL of enzyme solution. The reaction mixture was incubated at 37 °C for a period from 5–60 min before adding 10% TCA to terminate the reaction. The absorbance was read at 340 nm (Opert et al. 1997).

***Optimal pH determination of specific proteases***. Universal buffer (2 mM, pH range 3–14, Frugoni 1957) was used to obtain the optimal pH of each specific protease and find possible pH dependency of each substrate. The reaction mixtures were similar to those discussed above, but the pH of the buffer used in experimentation ranged from 3–14.

### Effect of mono- and di-valent cations on general proteolytic activity

Different concnetrations (1, 3, and 5 mM) of some selected ions including K^+^, Na^+^, Ca^2+^, Mn^+^, Mg^2+^, Zn^+^, and Fe^2+^ were used to obtain the potential alteration of general proteolytic activity in the salivary glands. Initially, 50 µL of each ion (different concentrations) was added to the universal buffer solution (2 mM, pH = 8) and gently stirred for 10 min. Then, 50 µL of 2% azocasein was added and stirred for and additional 10 min. After adding 20 µL of enzyme the solution was incubated for 60 min. Proteolysis was stopped by addition of 150 µL of 10% TCA. Precipitation was achieved by cooling to 4 °C for 120 min, and centrifugation occurred at 13,000 rpm for 10 min. An equal volume of 2 M NaOH was added to the supernatant, and absorbance was recorded at 450 nm. The blank solution consisted all mentioned portions except for the enzyme solution.

**Effect of general and specific inhibitors *General inhibitors***. Selected general inhibitors in this experiment consisted of sodium dodecylsulphate (SDS), urea, ethylendiamidetetraacetic acid (EDTA), and β-mercaptoethanol in concentrations of 1, 5, and 10 mM. Initially, 50 µL of each mentioned compound (different concentrations) was added to the universal buffer solution (2 mM, pH = 8) and gently stirred for 10 min. Then, 50 µL of 2% azocasein was added and stirred for an additional 10 min. After adding 20 µL of enzyme the solution was incubated for 60 min. Proteolysis was stopped by addition of 150 µL of 10% TCA. Precipitation was achieved by cooling to 4 °C for 120 min, and centrifuging at 10000 × g for 10 min. An equal volume of 2 M NaOH was added to the supernatant, and absorbance was recorded at 450 nm. The blank solution did not contain the enzyme.

***Specific inhibitors***. The following compounds were used to find alterations of the proteolytic activity in salivary glands in regards to specific substrates; soybean trypsin inhibitor (SBTI, 1, 3, and 5%), PMSF (phenylmethylsulfonyl fluoride, 1, 3, 5 mM); trypsin inhibitor, TLCK (Na-p-tosyl-L-lysine chlorom ethyl ketone, 1, 3, 5 mM); chymotrypsin inhibitor, TPCK (N-tosyl-Lphenylalanine chlorom ethyl ketone, 1, 3, 5 mM); cysteine protease inhibitor, E-64 (Ltrans-epoxysuccinyl-leucylamido-(4-guanidino)-butane, 1, 3, 5 mM); cystatin (1, 3, 5 mM) and metalloprotease inhibitors including phenanthroline, N, N, N′, N′tetraacetic acid (EGTA) and triethylenetetramine hexaacetic acid (TTHA). Also, DTT (dithiothreitol, 1, 3, 5 mM) as cysteine activator and elastatin (1, 3, 5 mM) as the inhibitor of elastase serine protease were used. Methods used to find specific inhibitor effects are described below.

### Electrophoresis zymogram

Electrophoretic detection (Laemmli 1970) of proteolytic enzyme was performed using resolving and stacking Polyacrylamide gels of 10% and 4%, respectively, according to the method described by Garcia-Carreno et al. (1993) with slight modifications. Nonreducing PAGE was carried out at 4 °C in a constant voltage of 110 mV so that gelatin (0.5%) was added in the resolving gel. When dye reached at the bottom of glace, the gel was carefully separated and put in universal buffer for 15 min. Gels were then washed in water and immediately fixed and stained with 0.1% Coomassie brilliant blue R-250 (AksoNobel Corporate, www.aksonobel.com) in methanol—acetic acid—water (50:10:40) overnight. Destaining was done in methanol— acetic acid— water (50:10:40) for at least two hours. Characterization of protease classes in SDS-PAGE zymograms using specific inhibitors was done according to Garcia-Carreno et al. (1993) with some modifications. Fifteen minutes before the experiment, 50 µL of the enzyme extract was mixed with 30 µL of inhibitors at 5 mM concentration including PMSF, TLCK and TPCK.

### Determination of endogenous trypsin inhibitors

Endogenous trypsin inhibitors from larvae of possible prey of *A. spinidens* were determined by a method described by Lwalaba et al. (2010) with slight modifications. The larvae used in this experiment consisted of *C. suppressalis, N. aenescens, P. brassicae, H. cunea*, and *E. kuhniella*. Two larvae with mechanically penetrated bodies were incubated in 500 mL of low glucose Ringer solution (121.5 mM NaCl; 10 mM KCl; 2.1 mM NaH_2_PO4; 10 mM NaHCO_3_; 0.7 mM MgCl_2_; 2.2 mM CaCl_2_; pH = 6.8 and 0.01 g glucose) for 30 min at 30 °C. Tissues were then ground with two types of (thin and thick) homogenizers. These samples from different species were heated to 90 °C for 30 min. Samples were centrifuged at 13,000 rpm for 20 min at 4 °C. The supernatant was transferred to new tubes and used as the source of trypsin inhibitor.

### Effect of endogenous trypsin inhibitor on trypsin-like protease activity

Initially, 50 µL of the inhibitors from the aforementioned larvae were added to universal buffer solution (2 mM, pH = 8) and gently stirred for 10 min. Then, 50 µL of BApNA (1 mM) was added and stirred for an additional 10 min. Incubation was initiated after adding 20 µL of enzyme for 20 min. 150 µL of 10% TCA was then added and absorbance was spectrophotometrically read at 405 nm by p-nitroaniline release. Electrophoresis zymogram was carried out as described above.

### Protein determination

Protein concentration was measured according to the method of Bradford (1976) using bovine serum albumin (Bio-Rad, www.biorad.com) as standard.

### Statistical analysis

The experimental design used completely randomized statistics. Tukey's and Factorial tests were used to compare data based on one-and two-way analyses. Statistical differences were considered at *p* ≤ 0.05 (SAS 1997).

## Results

### Salivary gland structure

**Figure 1.**
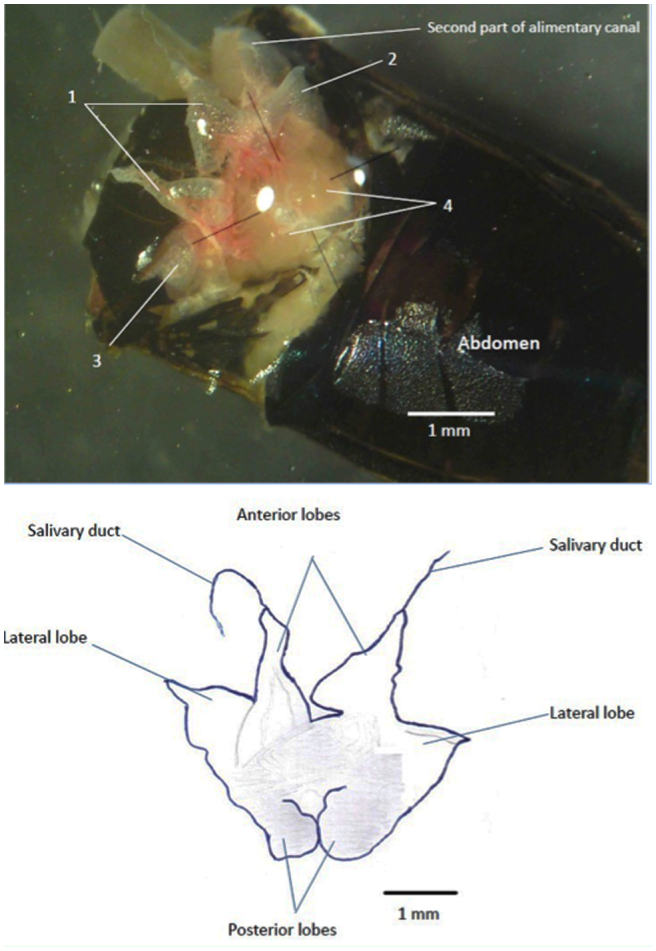
Salivary gland of the predatory bug, *Andrallus spinidens*. Image shows the salivary gland as a complex consists of four sections. Top: original image. Below: schematic image. 1 : Anterior lobes; 2,3: lateral lobes; 4: posterior lobes. High quality figures are available online.

Figure 1 shows the salivary glands of adult *A. spinidens*. Two anterior, two lateral, and two posterior lobes are present with two salivary ducts that transfer salivary secretions toward the stylet (Figure 1a, 1b).

### General proteolytic activity

**Figure 2.**
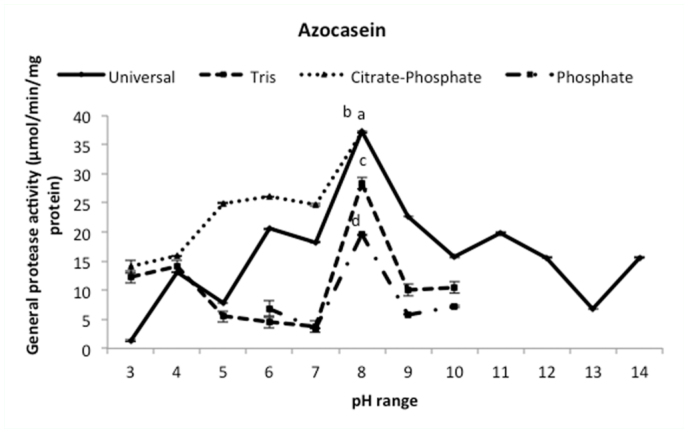
Optimal pH determination of the total proteolytic activity in the salivary gland of *Andrallus spinidens* by using different bufferic solution. Two-way analysis (Anova, Factorial test) was used to determine statistical differences showed by different letters (*p* ≤ 0.05). High quality figures are available online.

Samples prepared from salivary glands showed proteolytic activity by using 2% azocasein as the substrate (Figure 2). Proteolytic activity of salivary glands increased from pH 3 to 8; the highest proteolytic activity was found at pH 8 (Figure 2).

### Determination of optimal temperature (°C) on general proteolytic activity and stability

**Figure 3.**
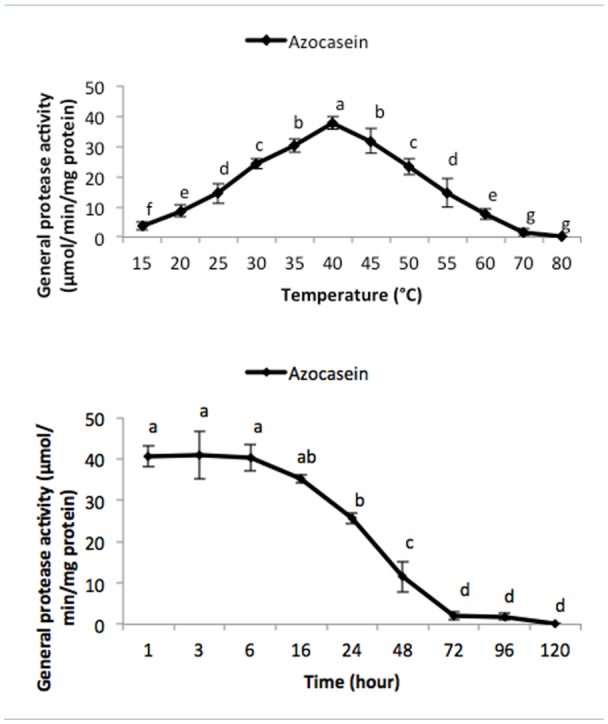
Optimal temperature (°C) determination of the total proteolytic activity in the salivary gland of *Andrallus spinidens* using Azocasein 2%. One-way analysis (Anova, Tukey's test) was used to determine statistical differences showed by different letters (*p* ≤ 0.05). Temperature stability (hour) of the total proteolytic activity in the salivary gland of *Andrallus spinidens* was carried out by using Azocasein 2% in different time intervals from 1 to 120 hours. One-way analysis (Anova, Tukey's test) was used to determine statistical differences showed by different letters (*p* ≤ 0.05). High quality figures are available online.

**Figure 4.**
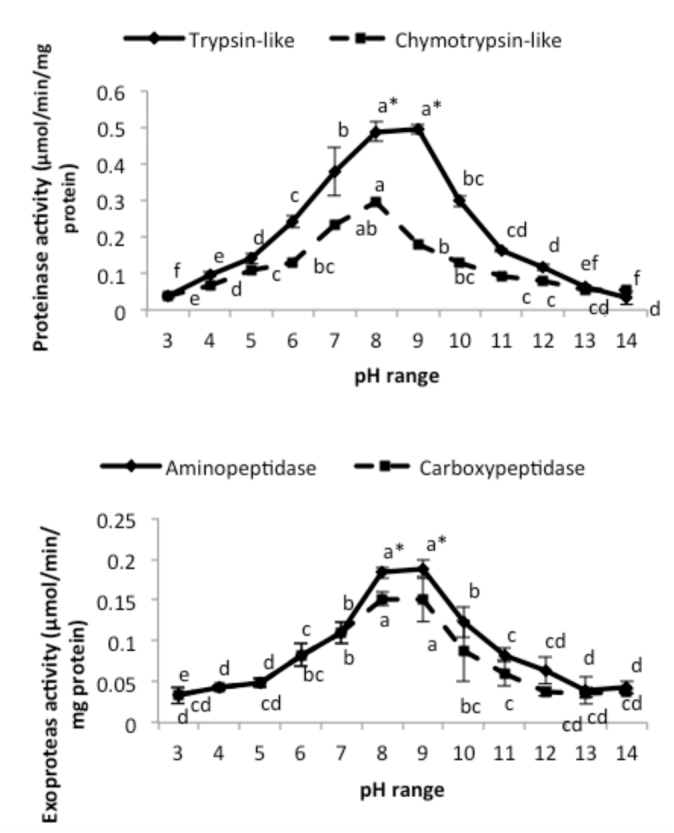
Optimal pH determination of the specific proteolytic activity in the salivary gland of *Andrallus spinidens* by using specific substrates. One-way analysis (Anova, Tukey's test) was used to determine statistical differences by various letters (*p* ≤ 0.05). Asterisks showed significant differences between optimal pH of two proteinase together and two exo-peptidase together (Anova, two-way analysis at *p* ≤ 0.05). High quality figures are available online.

General proteolytic activity increased from 15–40 °C using all three substrates in universal buffer, followed by a sharp decrease (Figure 3). The optimal temperature of the enzyme was found to be 40 °C using 2% azocasein (Figure 3). Also, the enzyme was stable for 6, 16, and 24 hours using 2% azocasein, hemoglobin, and casein, respectively (Figure 4).

### Specific proteolytic activity

Results showed tryspin-like, chymotrypsinlike, aminopeptidase, and carboxypeptidase activities in the salivary glands (Figure 6 and 7). Trypsin-like and aminopeptidase activities had the highest activity at pH 8 and 9, but chymotrypsin-like and carboxypeptidase showed the highest activity at pH 9 (Figure 6). Also, time course studies showed increasing slope of the line regression from 5 to 30 min, then slope decreased and the line plateaued (Figure 7). Linweaver-Burk plots revealed the highest *V_max_* value by using specific substrate of trypsin-like protease; the lowest value was found when carboxypetidase substrate was used (Figure 8, Table 1). Also, the lowest *K_m_*, values were found by using trypsin-, chymotrypsin-like protease, and carboxypeptidase activity; the highest value was found in the case of aminopeptidase substrate (Figure 8, Table 1).

### Effects of mono- and di-valent ions on general proteolytic activity

Table 2 shows the effect of some mono- and di-valent cations (concentrations of 1, 3, and 5 mM) on general proteolytic activity in adults of *A. spinidens*. Results revealed that Ca^2+^ (all concentrations) and Mg^2+^ (5 mM concentrations) were activators on the enzyme activity (Table 2). Other ions had the inhibitory effects on the general proteolytic activity so that Zn^+^ and Fe^2+^ had the highest and lowest inhibitory effects, respectively (Table 2). In addition, different concentrations of Na^+^ had no effect on enzyme activity (Table 2).

**Table 1.**
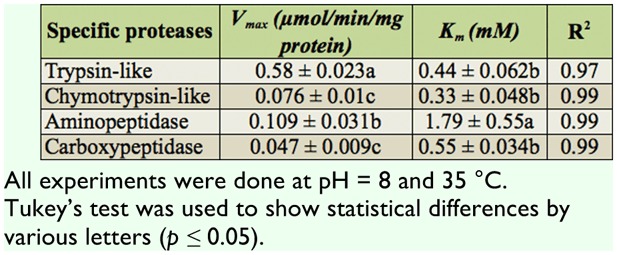
Kinetic parameters of specific proteolytic activity in the salivary glands of *Andrallus spinidens*.

**Table 2.**
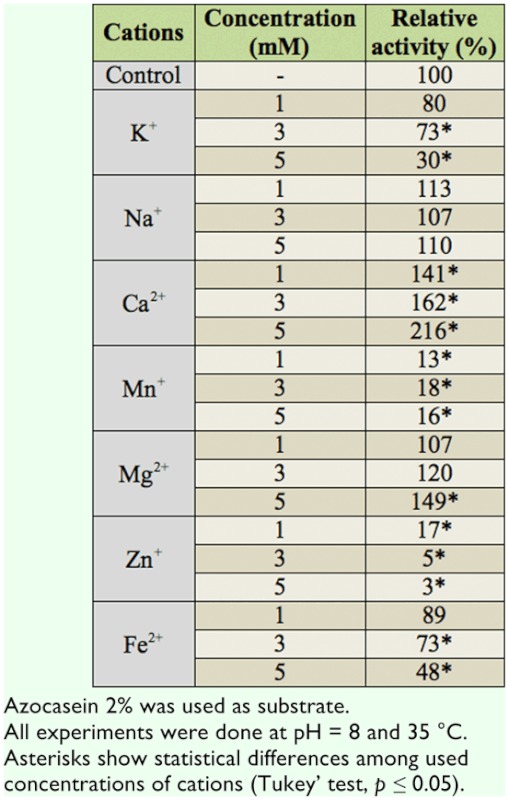
Effect of mono- and di-valent cations on proteolytic activity in the salivary glands of *Andrallus spinidens*.

**Effect of general and specific inhibitors *General inhibitors***. SDS, urea, EDTA, and βmercaptoethanol in 1, 5, and 10 mM concentrations caused inhibitory effects on the general proteolytic activity of *A. spinidens* (Table 3). In this case, urea and βmercaptoethanol showed the lowest and the highest effects on the enzymatic activity, respectively (Table 3). Also, EDTA significantly decreased the enzymatic activity, similar to β-mercaptoethanol (Table 3).

***Specific inhibitors***. Effects of specific inhibitors revealed that the assayed proteases in the salivary glands are serine protease and metalloproteinase (Table 4). It was found that SBTI and PMSF (as serine-protease inhibitors) significantly decreased enzymatic activity (Table 4). TLCK (as trypsin-like inhibitor) caused higher inhibition than TPCK (as chymotrypsin-like inhibitor) (Table 4). Phenanthroline, EGTA, and TTHA, as the metalloprotease inhibitor, calcium, and magnesium chelating agents, respectively, demonstrated presence of the metalloprotease in the salivary glands (Table 4). Using a specific chelating agent of calcium confirmed the presence of a calcium ion in the active site of the enzyme (Table 4). Other specific inhibitors such as elastatin, cystatine, DTT, and E-64 caused no significant effects on the proteolytic activity in the salivary glands (Table 4).

### Electrophoresis zymogram

Zymogram analysis of salivary proteases revealed four proteolytic bands in the control sample. Different classes of proteases were detected in the presence of the specific inhibitors by the disappearance or reduced intensity of the bands compared to the control. In this case, PMSF caused a strong inhibition on band P1 (21 kDa). Trypsin-like and chymotrypsin-like proteases were determined by zymography by using TLCK and TPCK due to the decrease of sharpness in band P1.

### Endogenous trypsin inhibitors

Endogenous tryspin inhibitors extracted from *C. suppressalis, N. aenescens, P. brassicae, H. cunea*, and *E. kuhniella* caused different effects on both assay and zymogram experiments. The results showed that the endogenous inhibitor from *C. suppressalis* had no effect on trypsin-like activity of salivary glands in *A. spinidens*. Endogenous inhibitors from *N. aenescens* and *H. cunea* similarly decreased trypsin-like protease activity, but *P. brassicae* and *E. kuhniella* strongly decreased the enzyme activity so that this inhibition, in the case of *P. brassicae*, was higher than in *E. kuhniella*.

**Table 3.**
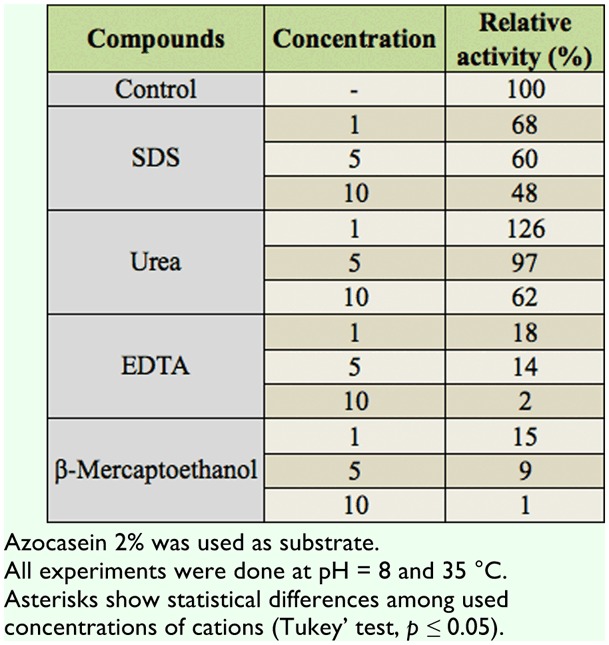
Effect of some general inhibitors on proteolytic activity in the salivary glands of *Andrallus spinidens*.

**Table 4.**
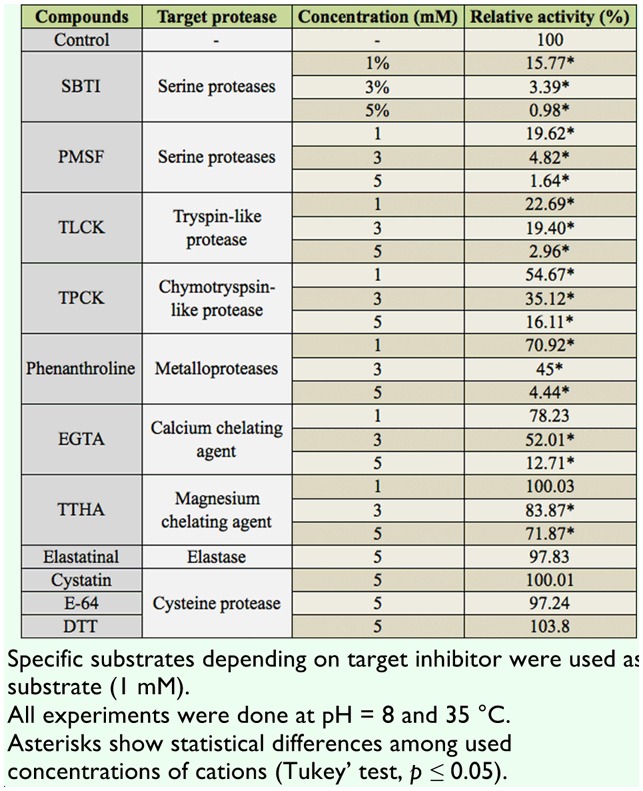
Effect of specific inhibitors on proteolytic activity in the salivary glands of *Andrallus spinidens*.

## Discussion

*A. spinidens* is a predator of rice pests in north of Iran especially because of synchronization of its life cycle with life cycles of rice pests such as *C. suppressalis* and *N. aenescens*. Studies on *A. spinidens* have been limited to its biology in rice fields and potential to decrease the population of rice pests (Najafi-Navaee et al. 1998; Mohaghegh and Najafi 2003). The current study was conducted to find the role of proteases as an important digestive enzymes in extra-oral digestion of its prey. Characterization of these enzymes in salivary secretions of *A. spinidens* could give an evolutionary background of predatory association of the bug with its prey. This fact depicts environmental effects such as temperature and agricultural techniques like spraying on the nature of salivary secretions. Of the four types of proteases, including trypsin-like, chymotrypsin-like, aminopeptidase, and carboxypeptidase, it was shown that trypsin-like protease had the highest enzymatic activity, although Cohen (1993) reported that aminopeptidase and carboxypeptidase were not found in the salivary glands but were found in the mid-gut. Additionally, our experiments to determine the optimal pH of proteolytic activity showed that pH 8 was optimal for both general and specific substrates, although the optimal pH of trypsin-like and aminopeptidase proteases were not statistically different at pH 8 and 9. The serine proteases, such as trypsin- and chymotrypsin-like proteases had peak activities at alkaline pH. Exopeptidases, such as amino and carboxypeptidases \ are known to be active at pH between 7–12. The results confirmed the optimal pH using four different types of buffer. Terra and Ferriera (2005) believed that enzyme pH optimum should be determined using different buffers to discount the effects of chemical constituents of the buffers and their ionic strength on enzyme activity. Bell et al. (2005) found the alkaline pH optimum and sensitivity to serine protease inhibitors, and thus concluded the existence of serine proeteolytic activity in the salivary secretions of *Podisus maculiventris*, a phenomenum reported also for *Zelus renardii* (Cohen 1993), *Creontiades dilutes* (Colebatch et al. 2002), *Lygus hesperus* (Zeng et al. 2002a), *Lygus lineolaris* (Zeng et al. 2002b), *Lethocerus uhleri* (Swart et al. 2006), *L. Hesperus* (Wright et al. 2006), and *P. nigripinus*. In the case of exopeptidases, Cohen (1993) showed that aminopeptidase and carboxypeptidase were not found in the salivary glands, but were instead found in the mid-gut.

The temperatures of 35 and 40 °C were optimal for activity of salivary proteases in *A. spinidens* using three general substrates. It was also found that the proteases were stable at these temperatures for 6–24 hours. These temperatures correspond to field temperatures during June–September when *A. spinidens* is active on Iranian rice fields. High activity of enzymes at a specific temperature of in vitro assays generally reflects the temperature of the environment where the organism feeds on the hosts. Extremes in temperatures can also disrupt the hydrogen bonds that hold the enzyme in its three-dimensional structure, leading denaturation of the protein (Zeng et al. 2000). In addition, biological reactions occur faster with increasing temperature, up to the point of enzyme denaturation, above which the enzyme activity and the rate of the reaction decreases sharply (Zeng et al. 2000). Oliveira et al. (2006) observed the highest activity of salivary proteases at 37.5 °C, but a smaller peak of activity was also evident at 25 ° C, suggesting the likely occurrence of different forms of trypsin-like proteases in the salivary glands of the predaceous bug, *P. nigrispinus*. Swart et al. (2006) demonstrated the highest proteolytic activity in the salivary secretion of two belostomatidae bugs, *Lethocerus uhleri* and *Belostoma lutarium* also at 37 °C.

Lineweaver-Burk analysis was used to find Vmax and Km values for general and specific proteolytic activity. Three general and four specific substrates revealed a higher *V_max_* value for azocasein, and a *K_m_*, value for casein in the case of general proteolytic activity, as well as for trypsin-like and chymotrypsin-like substrates. In the case of specific proteolytic activity, however, there were not any significant differences in the *K_m_*, value of trypsin-, chymotrypsin-like, and carboxypeptidase proteases. Linear plots directly calculate the values of *V_max_* (maximal velocity of the reactions) and *K_m_*, (The Michaelis constant) to give more information on the behavior or efficiency of the enzymes. Since the *K_m_* has an inverse relationship with the substrate concentration, saturating the active sites of the enzyme is required. In other words, *K_m_* is the measurement of the stability of the enzyme-substrate complex, and a low *K_m_* value would indicate strong binding. *V_max_* values show the breakdown rate of the enzyme-substrate complex so the higher *V_max_* value of trypsin-specific substrate demonstrated the higher enzymatic velocity (Zibaee and Bandani 2010).

In agricultural ecosystems, many fertilizers affect the presence of herbivores and carnivores. One of the side effects of these fertilizers could be enzymes that require ions in their active site. In fact, ions—especially di-valent ions—work as cofactors and increase enzymatic activity. In our experiments, proteolytic activity in salivary secretions of *A. spinidens* increased in the presence of the divalent ions Mg^2+^ and Ca^2+^. To prove this finding, two specific chelating agents, EGTA (Ca^2+^ chelating agent) and TTHA (Mg^2+^ chelating agent), a general chelating agent, EDTA, and phenanthroline, the specific inhibitor of metalloproteases, were used. Our results showed that EDTA significantly decreased the proteolytic activity as well as EGTA and TTHA although the inhibition caused by EGTA was higher than that of TTHA. These finding demonstrated the importance of Ca^2+^ ions in the active site of the enzyme. Meanwhile, inhibition of the enzyme by TTHA suggests the necessity of binding of some magnesium atoms in the activation center of *A. spinidens* proteases as was found by Feng et al. (2008). On the other hand, the stimulation of proteolytic activity by equimolar concentrations of Ca^2+^ and Mg^2+^ ions suggest that one could substitute for the other during activation (Feng et al. 2008).

Different specific protease inhibitors including SBTI, PMSF, TLCK, TPCK, Elastatin, Cystatin, E-64, and DTT were used to confirm the type of proteases present in the salivary glands of *A. spinidens*. SBTI and PMSF greatly inhibited enzymatic activity, demonstrating the presence of serine proteases in salivary glands. TLCK (trypsin-like inhibitor) and TPCK (chymotrypsin-like inhibitor) decreased enzymatic activity in different proportions, demonstrating the more integral role of trypsin-like proteases. Zhu et al. (2003) studied the effects of 11 specific inhibitors on salivary proteolytic activity of *L. lineolaris*. The general protease activity in the salivary glands was effectively suppressed by several inhibitors. Among the inhibitors tested, aprotinin and benzamidine exhibited the highest inhibition (80%) against azocaseinase activity in the salivary glands, followed by PMSF (74%), PCPI (65%), and STI (62%). Leupeptin showed moderate inhibition (49%), followed by E-64 and TLCK (∼30%). Bell et al. (2005) reported that the serine proteinase inhibitor (PMSF) caused the most marked reduction in the rate of proteolysis, with a residual activity of approximately 53% in the saliva of *P. maculiventris*. Also, they found no inhibition of the plant-derived serine proteinase inhibitors (CpTI and SKTI), with the exception of SBTI (Bell et al. 2005). Oliveira et al. (2006) demonstrated that the proteases present in salivary gland extracts of *P. nigrispinus* were able to hydrolyse the serine protease substrate L-BApNA, and were inhibited by TLCK and benzamidine, two classical trypsin-like inhibitors, providing the evidence of a major involvement of trypsinlike proteases in the extra-oral digestion of *P. nigrispinus*. Proteolytic activity in the saliva of *B. lutarium* and *L. uhleri* was reduced by the protease inhibitors PMSF, TLCK, and TPCK. However, although activity was reduced, significant proteolytic activity remained in the presence of either TLCK or TPCK independently (Swart et al. 2006). Using TPCK and TLCK concurrently eliminated most protease activity in saliva of *B. lutarium*, but did not significantly increase the inhibition of activity in *L. uhleri* saliva when compared to TLCK or TPCK alone (Swart et al. 2006). Wright et al. (2006) found the presence of both trypsin- and chymotrypsin-like activities in the salivary glands of *L. hesperus* by using specific substrates and inhibitors. They concluded that these results might be due to its zoophytophagous feeding strategy, or indicative of the digestive plasticity of this polyphagous insect. Meiser et al. (2010) found no inhibitory effects of PMSF, TLCK, and TPCK on salivary proteases of *Panstrongylus megistus*, but other serine protease inhibitors such as antipain, benzamidine, soybean trypsin inhibitor, aprotinin, and APMSF (4-amidinobenzylsulphonyl fluoride hydrochloride) decreased the proteolytic activity between 38–77%. The results of these mentioned studies correspond to our results on inhibitory effects of serine protease inhibitors on proteolytic activity of salivary glands in *A. spinidens*. Class-specific inhibitors are useful tools to identify types of proteases as well as their substrate specificity. Direct measurement of class specific proteolytic activities using combinations of inhibitors to inhibit all but one class of protease may aid in the accurate classification and quantification of digestive proteases, especially against large protein substrates (Wright et al. 2006).

Two caseinolytic proteinases from salivary glands of *L. lineolaris*, approximately 26 and 30 kDa, were detected by Wright et al. (2003). Meiser et al. (2010) used SDS-PAGE of proteins obtained after anion exchange chromatography and found two bands with molecular weights of 39 and 30 kDa under nonreducing conditions, and 34 and 27 kDa under reducing conditions in *P. megistus*.

Five endogenous tryspin inhibitors from *C. suppressalis, N. aenescens, P. brassicae, H. cunea*, and *E. kuhniella* had various effects on trypsin-like activity in the salivary glands of *A. spinidens*. The highest inhibitory effect was observed in the case of *P. brassicae*, and no statistical difference was obtained on *C. suppressalis* glands compared to the control. This is an interesting co-evolutionary point especially in the case *C. suppressalis. Andrallus spinidens* is known as a potential predator of *C. suppressalis*. The larvae of *C. suppressalis* damage rice production each year in the north of Iran, and they are an available prey for *A. spinidens*. Thus, predaceous bugs may overcome enzymatic inhibitory effects of *C. suppressalis*, and utilize them as a food source. Another rice pest, *N. aenescens*, is a periodic pest of rice and has population outbreaks every 4–5 years, and thus is not regularly available for *A. spinidens*. Many endogenous protease inhibitors have been extracted from various insect tissues (blood, ovaries, and fat body) (Hamdaoui et al. 1998; Kanost 1999; Simonet et al. 2002). We found that boiled extracts inactivated endogenous enzymes, up to 80% inhibition in the case of protease. Endogenous inhibitors can protect insects from bacteria and fungi that enter the alimentary canal along with food. These inhibitors may have dual roles by decrease the proteolytic activity in saliva secretion of the predator and cause damage to self-protease on tissues.

Extra-oral digestion in predacious bugs such as *A. spinidens* facilitates their ability to utilize larger insects, but requires a relatively long period of time with their prey before satiation; it takes between three and four hours for *A. spinidens* to eat a larvae of *C. suppressalis* completely (personal observation). This reflects the considerable investment of *A. spinidens* makes in production of enzymes that are injected into the prey, waiting for the enzymes to act, and recovering the digested products (Oliveira et al. 2006).

**Figure 5.**
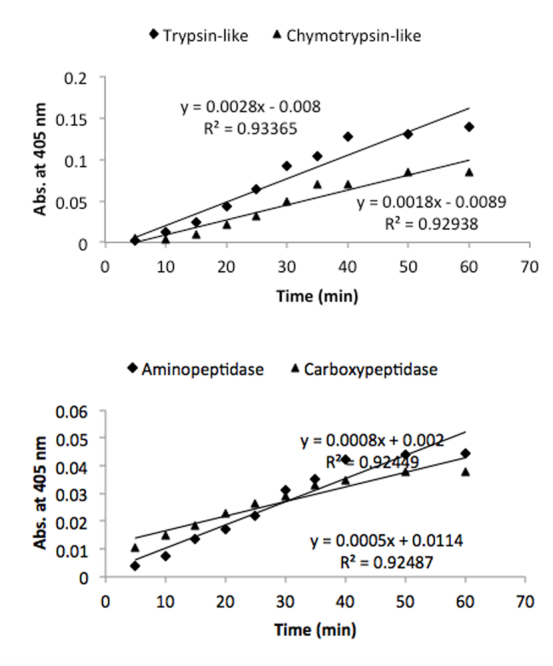
Time course determination of the specific proteolytic activity in the salivary glands of *Andrallus spinidens*. First, 80 µL appropriate buffer (in all range) incubated with 20 µLsubstrate at 35 °C as a standard temperature in enzymatic activity. After 10 min, 7 µL of enzyme added and allowed the reaction to continue for 60 min. At each time interval, absorbance was read at 405 nm. High quality figures are available online.

**Figure 6.**
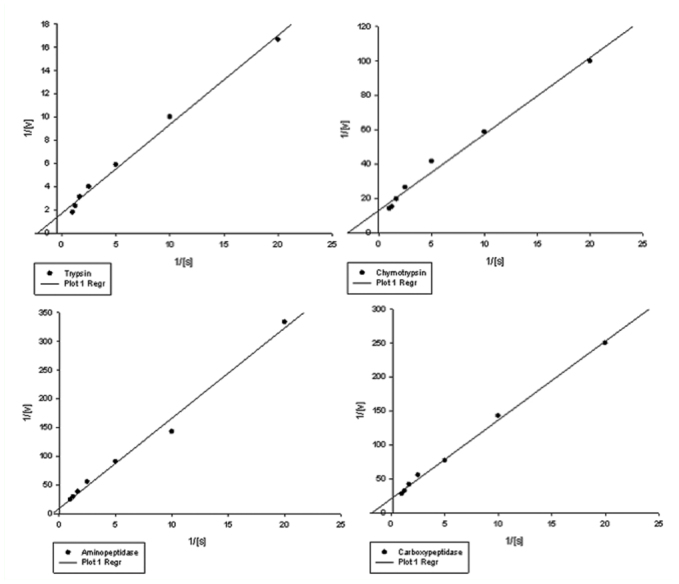
Double reciprocal plot to show the kinetic parameters of the specific proteolytic activity in salivary gland of *Andrallus spinidens* (1/V_max_ = intercept on the 1/V_0_ ordinate, -1/Km= intercept on the negative side of the 1/[S] abscissa). Different concentrations (0.1, 0.3, 0.5, 0.8 and 1 mM) of each substrate prepared and used in the kinetic parameter experiments. High quality figures are available online.

**Figure 7.**
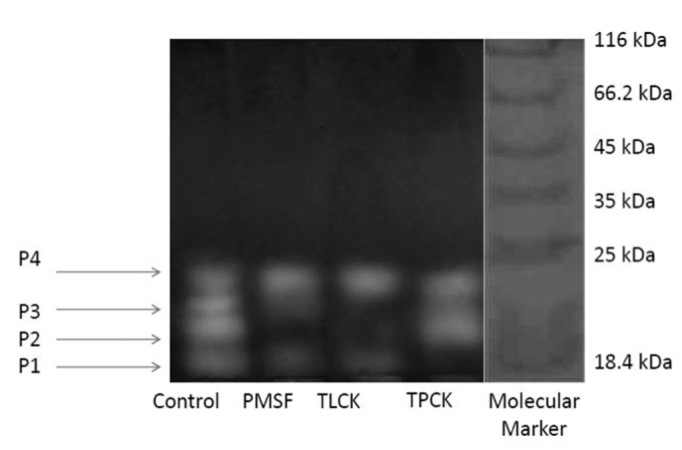
Zymogram analysis of the effect of proteinase inhibitors on casein hydrolytic activity in the salivary glands of *Andrallus spinidens*, by using 10% non-reducing SDS-PAGE. MWM: molecular weight markers (kDa). Control: no inhibitor, PMSF 5mM, TLCK 5mM, TPCK 5mM. High quality figures are available online.

**Figure 8.**
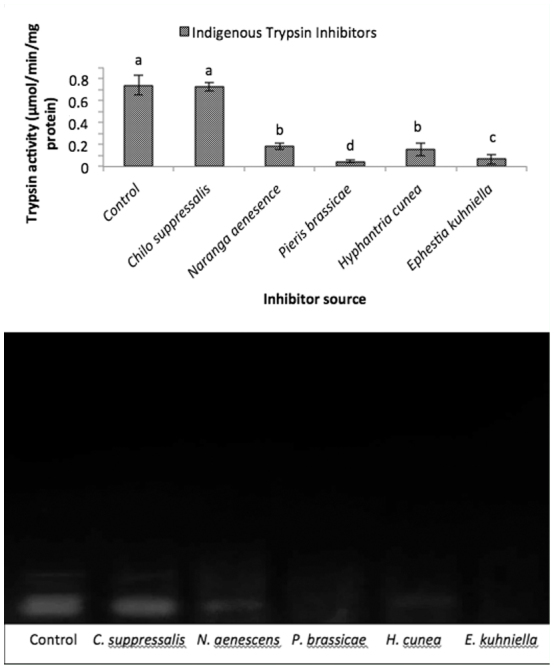
Indigenous inhibitory effects of different lepidopteran larvae on trypsin activity in the salivary glands of *Andrallus spinidens*. After initial incubation of 100 µL buffer (Universal, pH = 8) with trypsin specific substrate (30 µL) for 10 min, extracted indigenous inhibitor of each mentioned larvae added and the mixture incubated for additional 10 min. Then, 20 µL of enzyme added and absorbance was read at 405 nm after 20 min. For zymogram analysis, resolving gel (10%) containing gelatin and stacking one (4%) was prepared and samples were loaded to each well. After reaching dye bottom of the gel, gel gently removed from glasses, rinsed in staining solution overnight. Then it was destained and white bands were observed at dark blue background. One-way analysis (Anova, Tukey's test) was used to determine statistical differences by various letters (*p* ≤ 0.05). High quality figures are available online.
